# The Risk of Penetration–Aspiration Related to Residue in the Pharynx

**DOI:** 10.1044/2020_AJSLP-20-00042

**Published:** 2020-06-29

**Authors:** Catriona M. Steele, Melanie Peladeau-Pigeon, Emily Barrett, Talia S. Wolkin

**Affiliations:** a Swallowing Rehabilitation Research Laboratory, KITE, Toronto Rehabilitation Institute, University Health Network, Canada; b Department of Speech-Language Pathology, Rehabilitation Sciences Institute, Faculty of Medicine, University of Toronto, Canada

## Abstract

**Purpose:**

Reference data from healthy adults under the age of 60 years suggest that the 75th and 95th percentiles for pharyngeal residue on swallows of thin liquids are 1% and 3%(C2-4)^2^, respectively. We explored how pharyngeal residue below versus above these values prior to a swallow predicts penetration–aspiration.

**Method:**

The study involved retrospective analysis of a previous research data set from 305 adults at risk for dysphagia. Participants swallowed six thin boluses and three each of mildly, moderately, and extremely thick barium in videofluoroscopy. Raters measured preswallow residue in %(C2-4)^2^ units and Penetration–Aspiration Scale (PAS) scores for each swallow. Swallows were classified as (a) “clean baseline” (with no preswallow residue), (b) “clearing” swallows of residue with no new material added, or (c) swallows of “additional material” plus preswallow residue. Frequencies of PAS scores of ≥ 3 were compared across swallow type by consistency according to residue severity (i.e., ≤ vs. > 1%(C2-4)^2^ and ≤ vs. > 3%(C2-4)^2^.

**Results:**

The data set comprised 2,541 clean baseline, 209 clearing, and 1,722 swallows of additional material. On clean baseline swallows, frequencies of PAS scores of ≥ 3 were 5% for thin and mildly thick liquids and 1% for moderately/extremely thick liquids. Compared to clean baseline swallows, the odds of penetration–aspiration on thin liquids increased 4.60-fold above the 1% threshold and 4.20-fold above the 3% threshold (mildly thick: 2.11-fold > 1%(C2-4)^2^, 2.26-fold > 3%(C2-4)^2^). PAS scores of ≥ 3 did not occur with clearing swallows of moderately/extremely thick liquids. Lower frequencies of above-threshold preswallow residue were seen for swallows of additional material than for clearing swallows. Compared to clean baseline swallows, the odds of PAS scores of ≥ 3 on swallows of additional material increased ≥ 1.86-fold above the 1% threshold and ≥ 2.15-fold above the 3% threshold, depending on consistency.

**Conclusion:**

The data suggest that a pharyngeal residue threshold of 1%(C2-4)^2^ is a meaningful cut-point for delineating increased risk of penetration–aspiration on a subsequent swallow.

Oropharyngeal dysphagia involves impairments in airway protection and/or bolus clearance (Clavé & Shaker, 2015), which manifest as penetration–aspiration and/or pharyngeal residue. Although these problems can co-occur in a single patient on the same swallow (e.g., Simon et al., 2020), they are thought to have different underlying mechanisms (see Curtis et al., 2020; Leonard et al., 2011; Vose & Humbert, 2019; Waito, Steele, et al., 2018; Waito, Tabor-Gray, et al., 2018). Across the literature, pharyngeal residue is frequently mentioned as a risk for subsequent aspiration (e.g., Brodsky et al., 2010; Hadjikoutis et al., 2000; Nishino & Hiraga, 1991; Preiksaitis & Mills, 1996). However, thresholds of residue severity that predict subsequent airway invasion are yet to be clearly established. The goal of the current study was to address this knowledge gap by calculating the risk of airway invasion on swallows of different liquid consistencies, as a function of the amount and location of preexisting residue in the pharynx (henceforth “preswallow residue”).

Molfenter and Steele (2013) retrospectively analyzed videofluoroscopy recordings of thin liquid barium for cases where a person swallowed more than once for a single bolus. They used the Normalized Residue Ratio Scale (NRRS; Pearson et al., 2013) to explore thresholds of residue severity in the valleculae and the pyriform sinuses that predicted scores of 3 or higher on the Penetration–Aspiration Scale (PAS; Rosenbek et al., 1996) on subsequent, noninitial swallows of the same bolus. These subsequent swallows were limited to “clearing” swallows, meaning that piecemeal swallows (in which additional material was added from the oral cavity) were excluded. Molfenter and Steele (2013) identified an NRRS threshold in the valleculae of ≥ 0.09, at which the risk of penetration–aspiration on the next swallow increased 2.07-fold compared to residue below this value. They were, however, unable to identify a similar threshold for pyriform sinus residue.

More recently, Namasivayam-MacDonald and Riquelme (2019) explored the relationship between residue and subsequent penetration–aspiration with thin and extremely thick liquids in adults with dementia. The NRRS thresholds chosen by Namasivayam-MacDonald and Riquelme came from recently published descriptive statistics for thin liquid residue in healthy young adults (Steele, Peladeau-Pigeon, et al., 2019). Using the upper 95% confidence interval (CI) values from those data, NRRS thresholds of > 0.04 for the valleculae and > 0.01 for the pyriform sinuses were studied. The odds of PAS scores of ≥ 3 on subsequent swallows of thin or extremely thick liquid were calculated, relative to these thresholds. Unlike Molfenter and Steele (2013), Namasivayam-MacDonald and Riquelme did not differentiate between type of noninitial swallow, including both clearing and piecemeal swallows in their analysis. In contrast to the results reported for clearing swallows by Molfenter and Steele, they found no relationship between vallecular residue exceeding an NRRS of 0.04 and penetration–aspiration on the subsequent swallow, with either consistency. However, with thin liquids, pyriform sinus residue above the NRRS threshold of 0.01 led to a 2.83-fold increase in the odds of penetration–aspiration on the subsequent swallow.

Both of these previous studies limited their focus to penetration–aspiration on noninitial swallows of thin or extremely thick liquid boluses (i.e., second or higher swallows of a bolus in cases where there was more than one swallow for a bolus). However, it is false to assume that initial swallows of new boluses always begin with “clean baseline” conditions (i.e., with no preswallow residue in the pharynx). In fact, clean baseline conditions can only be assumed for the very first bolus presented in an assessment or research protocol, and even here, pooled secretions may be present but not visible on videofluoroscopy (Miles et al., 2018; Murray et al., 1996). In reality, after the initial swallow of the first bolus in a protocol, there is always the possibility that residue from previous swallows may be present, contaminating the starting conditions for all subsequent swallows and boluses. Additionally, in cases where there is a loss of oral bolus control and premature spill of material into the pharynx, it is possible that the material may already be in the pharynx prior to volitional transfer of a bolus, even on the very first bolus in a protocol. Recognizing these possibilities, we decided to examine the risk of penetration–aspiration on any swallow, relative to the presence/absence and amount of residue seen on videofluoroscopy at the beginning of that swallow.

Steele and colleagues (Steele, Peladeau-Pigeon, et al., 2019; Steele et al., 2020) have recently recommended the use of anatomically referenced pixel-based measurements in %(C2-4)^2^ units for valid, reliable, and precise measurement of pharyngeal residue. Unlike the NRRS, the %(C2-4)^2^ equation does not require measurement of the area of the space where the residue is located (i.e., either the valleculae or the pyriform sinuses). Because the %(C2-4)^2^ equation is not linked to a particular anatomical space, it offers the opportunity to measure residue in the valleculae, pyriform sinuses, and elsewhere in the pharynx and also enables the calculation of a composite impression of total pharyngeal residue through summation of measures across all locations. Web-based resources from the Steele lab (https://steeleswallowinglab.ca/srrl/best-practice/vfss-analysis/) point out that distributions of residue are typically positively skewed; consequently, upper boundaries for the healthy reference range are best delineated by the 95th percentile, and values above the 75th percentile can be considered to fall in an at-risk zone that approaches abnormal (Ceriotti et al., 2009; Ozarda, 2016). The 75th and 95th percentile boundaries for total pharyngeal residue in healthy adults under the age of 60 years, after an initial swallow of thin liquid, fall at 1% and 3%(C2-4)^2^, respectively. We adopted these thresholds for classifying residue severity.

The objective of this study was to examine the frequency and odds of penetration–aspiration on swallows of different liquid consistencies as a function of the presence of above- versus below-threshold preswallow residue. Consistent with previous studies (Molfenter & Steele, 2013; Namasivayam-MacDonald & Riquelme, 2019; Steele, Mukherjee, et al., 2019), we defined penetration–aspiration events of concern as those with PAS scores of 3 and higher (henceforth referred to as “unsafe” or “penetration–aspiration”). We defined new boluses as new sips or spoonfuls of material. Initial swallows were defined as the first swallow of a new bolus, and noninitial swallows were the second or higher swallow in cases where more than one swallow was seen for a bolus. Both initial and noninitial swallows were classified further into three types, as follows:

1. swallows with “clean baseline” conditions (i.e., no preswallow residue present);
2. “clearing swallows” of preswallow pharyngeal residue, without the addition of new material from the mouth; and
3. “swallows of additional material” on top of preswallow pharyngeal residue.

To further explain these classifications, it is worth noting the following:

- Clean baseline swallows would generally be expected for the initial swallow of the first bolus in a protocol, except in cases where premature spillage of that bolus into the pharynx has occurred prior to the onset of fluoroscopy, such that material is already visible in the pharynx on the very first frame of recording;
- clean baseline swallows might be seen on initial swallows of new boluses when the pharynx has previously been cleared of all residue;
- clean baseline conditions might be seen on noninitial swallows of boluses where the bolus is divided into more than one portion in the mouth, and transfer of a second or higher portion of that bolus occurs after complete pharyngeal clearance of an earlier portion;
- clearing swallows, which do not involve the transfer of new material from the mouth to the pharynx, would only be expected to occur on noninitial swallows;
- the “swallows of additional material” classification would apply to noninitial swallows of a bolus where preceding swallows of that same bolus have left residue in the pharynx; and
- situations where residue from one or more previous swallows is present in the pharynx prior to the initial swallow of a new bolus would also be classified as swallows of additional material.

## Method

This article involves secondary analysis of a data set from a previous study (Steele, Mukherjee, et al., 2019) comprising videofluoroscopy recordings for 15 boluses per participant in 305 adults considered to be at risk for dysphagia. Human subjects approval for secondary analysis of the data set was obtained from the local institutional research ethics board. As reported in the original article, the participants were adults with diagnoses of stroke or acquired brain injury, or other inpatients or outpatients with signs or symptoms of dysphagia aged ≥ 50 years. Individuals with history of head and neck cancer, known congenital or structural abnormalities in the oropharynx, or major surgery to the mouth and neck were excluded. The protocol began with six naturally sized sips of a thin, 20% w/v barium suspension (Bracco Diagnostics, Inc., Varibar Thin, diluted with water), followed by three sips of mildly thick and three teaspoons each of moderately thick and extremely thick barium. The thickened stimuli were prepared by adding Nestlé Resource ThickenUp Clear xanthan gum thickener to the 20% w/v thin barium recipe. Additional details regarding data collection can be found in the original article and its appendix (Steele, Mukherjee, et al., 2019).

### Data Processing and Rating

For the original study, the videofluoroscopy recordings were transferred to a core lab where they were spliced into shorter clips without audio, containing one bolus per clip. In cases where more than one swallow was performed for a single bolus, all swallows for that bolus were contained in the same clip. Rating was completed in duplicate by trained raters according to a standard operating procedure, which began with counting the number of swallows for each bolus and rating swallowing safety using the PAS (Rosenbek et al., 1996) for every swallow. These PAS scores were then converted into binary classes of “safe” (scores of 1 and 2) and “unsafe” (scores of 3 and higher). The “swallow rest” frame at the end of each swallow was also identified (defined as the first frame showing the pyriform sinuses at their lowest position, relative to the spine, prior to onset of the hyoid burst for a subsequent swallow), and pixel-based measurements of vallecular and pyriform sinus residue were taken on those swallow rest frames. Measures of residue elsewhere in the pharynx were not included. Raters were blind to bolus consistency and to the order of bolus presentation. Disagreements in rating were resolved by consensus.

For the current article, the binary PAS classifications from the original study were used. Additional secondary analyses involved the following steps:

1. Each swallow was classified as “initial”/”noninitial” (per bolus);
2. each swallow was classified by type (i.e., “clean baseline,” “clearing swallow,” or “additional material”);
3. for initial swallows, a “preswallow” frame was identified, prior to the movement of new material from the mouth to the pharynx, and preswallow vallecular and pixel-based measures of pyriform sinus residue were made on that frame; and
4. for noninitial swallows, vallecular and pyriform sinus residue measurements from the swallow rest frame of the preceding swallow of the same bolus were extracted from the original data set to be used as measures of preswallow residue.

All pixel-based measures of residue were expressed in %(C2-4)^2^ units, and the vallecular and pyriform sinus residue measures were added together for a composite “sum vallecular and pyriform sinus residue” measure. Thirty percent of the preswallow residue measurements were performed in duplicate for the purposes of evaluating interrater agreement. Intraclass coefficients showed excellent interrater reliability (ICC = .9, 95% CI [.89, .92]).

## Analysis

The frequencies of initial/noninitial swallows and swallow type were tabulated by consistency and bolus number in the protocol. Descriptive statistics for preswallow residue severity in %(C2-4)^2^ units were calculated for the clearing swallows and swallows of additional material by consistency (range, median, and percentile values). The frequencies of swallows with above- versus below-threshold preswallow residue were cross-tabulated by swallow type (clean baseline swallows, clearing swallows, swallows of additional material), the location of above-threshold residue (isolated to the valleculae, isolated to the pyriform sinuses, or present in both locations), and consistency. The 1% and 3%(C2-4)^2^ thresholds of preswallow residue severity were explored separately. The frequencies of PAS scores of < 3 versus ≥ 3 were then cross-tabulated by swallow type and residue severity classification (i.e., above or below threshold). Odds ratios for penetration–aspiration were calculated between the above- and below-threshold residue classes. Finally, to determine the point where preswallow residue begins to emerge as a risk for penetration–aspiration on thin liquid swallows, we undertook post hoc iterative exploration of residue thresholds in 0.5%(C2-4)^2^ increments below 1%(C2-4)^2^ until the apparent breakpoint between neutral and increased odds was identified.

## Results

The data set included a total of 3,590 boluses and 4,472 swallows. Table 1 shows the number of swallows in the data set broken down by swallow type, initial versus noninitial swallows, bolus number, and consistency. It should be noted that the consistency label refers to the consistency of the bolus being swallowed, rather than the presumed consistency of any preswallow residue present. The only exception to this is for clearing swallows, where there is no new bolus; here, the consistency label refers to the known consistency of the bolus swallowed in the preceding swallow.

**Table 1.** Swallows available in the data set, by consistency, swallows per bolus, swallow number, and swallow type.

Consistency	Bolus no. in protocol	No. of swallows per bolus	Swallow no. (per bolus)	Swallow type (count, %^a^)		
				Clean baseline	Clearing	Additional material
Thin	1	1	1st (initial)	185 (9)	N/A	7^b^ (0.3)
		More than 1	1st (initial)	62 (3)	N/A	6^b^ (0.3)
			2nd or higher (noninitial)	68 (3)	33 (2)	15 (0.7)
	2–6	1	1st (initial)	584 (29)	N/A	354 (17)
		More than 1	1st (initial)	149 (7)	N/A	145 (7)
			2nd or higher (noninitial)	235 (11)	90 (4)	117 (6)
Mildly thick	7–9	1	1st (initial)	329 (34)	N/A	272 (28)
		More than 1	1st (initial)	20 (2)	N/A	70 (7)
			2nd or higher (noninitial)	110 (12)	51 (5)	107 (11)
Moderately thick	10–12	1	1st (initial)	307 (43)	N/A	252 (35)
		More than 1	1st (initial)	15 (2)	N/A	32 (5)
			2nd or higher (noninitial)	26 (4)	21 (3)	36 (5)
Extremely thick	13–15	1	1st (initial)	344 (48)	N/A	237 (32)
		More than 1	1st (initial)	15 (2)	N/A	26 (4)
			2nd or higher (noninitial)	51 (7)	14 (2)	46 (6)
Total	All boluses	All single swallows	All 1st (initial)	1,790 (40)^c^	N/A	1,122 (25)^c^
		All multiple swallows	All 1st (initial)	291 (7)^c^	N/A	325 (7)^c^
			All 2nd or higher (noninitial)	460 (10)^c^	209 (5)^c^	275 (6)^c^
		All swallows		2,541 (57)^c^	209 (5)^c^	1,722 (39)^c^

*Note.* N/A = not applicable.

a Percentages are shown by consistency and are rounded to the nearest percent unless the numbers fell below 1%.

b Barium visible below the ramus of mandible on first frame of video recording.

c Percentages of the total data set of 4,472 swallows, rounded to the nearest percent.

**Figure 1.**
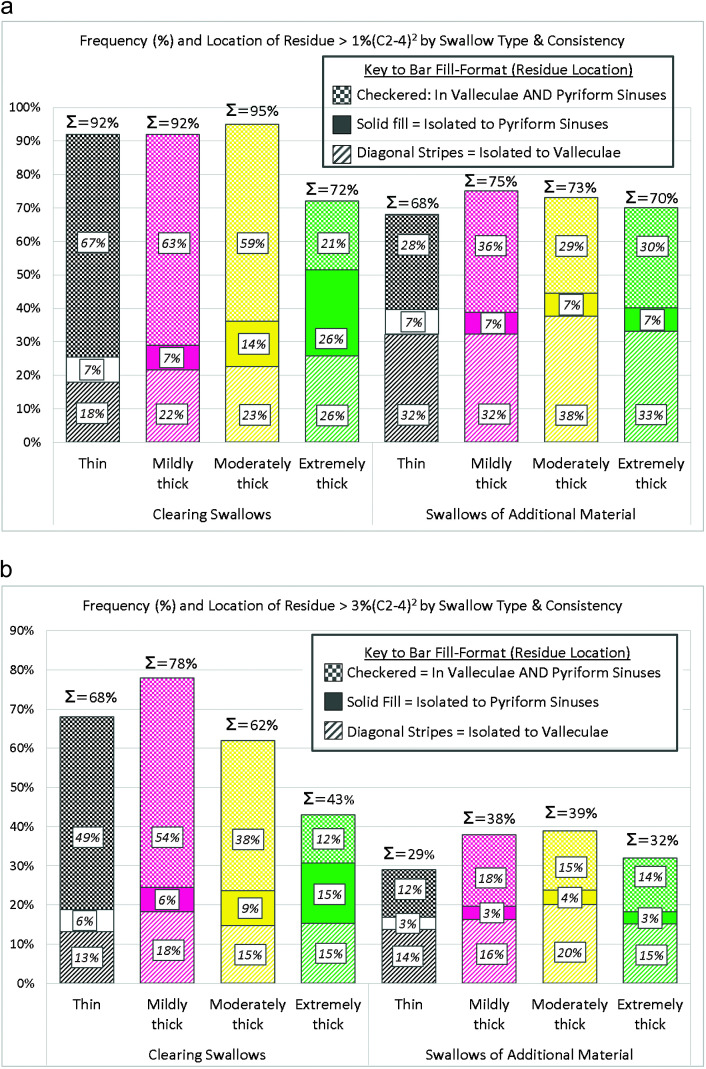
(a) Frequencies of preswallow residue above 1%(C2-4)^2^ arranged by consistency for the clearing swallows and the swallows of additional material. Percentage frequencies across all pharyngeal locations are shown above each bar. The subdivisions of each bar indicate the location of the above-threshold preswallow residue, with frequencies shown in italic font. (b) Frequencies of preswallow residue above 3%(C2-4)^2^ arranged by consistency for the clearing swallows and the swallows of additional material. Percentage frequencies across all pharyngeal locations are shown above each bar. The subdivisions of each bar indicate the location of the above-threshold preswallow residue, with frequencies shown in italic font.

Table 2 provides descriptive statistics for combined vallecular and pyriform preswallow residue severity for the clearing swallows and swallows of additional material. Values for residue present prior to clearing swallows were higher than those preceding swallows of additional material up to the 95th percentile. Figures 1a and 1b illustrate the frequencies of above-threshold preswallow residue for the clearing swallows and the swallows of additional material, beginning with the > 1%(C2-4)^2^ threshold and followed by the > 3% threshold. Consistent with the previous observation regarding residue severity, it can be appreciated from these figures that preswallow residue above either threshold was more common for clearing swallows than for swallows of additional material. The subdivisions of the bars in Figures 1a and 1b indicate the location of the above-threshold preswallow residue. Isolated preswallow residue exceeding either threshold was much less common in the pyriform sinuses (solid shaded subdivisions) than in the valleculae (diagonal dashed subdivisions) or distributed across both locations (checkered subdivisions). When the percentage frequencies within each swallow type were multiplied by the number of cases of each swallow type (see Table 1), this translated to a very small number of cases of isolated above-threshold preswallow pyriform sinus residue. For this reason, the subsequent calculations of odds ratios for penetration–aspiration were performed using only the composite “sum vallecular and pyriform sinus residue” measure rather than stratified by location.

**Table 2.** Descriptive statistics for preswallow pharyngeal residue (summed across the valleculae and pyriform sinuses) in %(C2-4)^2^ units, by swallow type.

Swallow type	Consistency	Minimum	25th %ile	*Mdn*	75th %ile	95th %ile	Maximum
Clearing swallows	Thin	0.31	2.65	4.94	9.15	14.98	20.68
	Mildly thick	0.73	3.57	6.01	11.50	16.32	20.19
	Moderately thick	0.79	2.13	6.37	10.99	17.72	17.96
	Extremely thick	0.59	0.82	2.53	6.63	N/A	23.52
Swallows of additional material	Thin	0.06	0.82	1.64	3.41	9.98	22.38
	Mildly thick	0.06	0.97	2.14	4.81	11.15	26.04
	Moderately thick	0.08	0.94	2.08	4.52	11.63	32.09
	Extremely thick	0.13	0.88	1.81	3.68	10.72	26.82

*Note.* N/A = not available.

Table 3 shows the frequencies of penetration–aspiration by swallow type and consistency. On clean baseline swallows, frequencies of penetration–aspiration were 5% for thin and mildly thick liquids and 1% for moderately and extremely thick liquids. Table 4 shows a further breakdown by swallow type, in situations where preswallow residue exceeded the 1% and 3%(C2-4)^2^ thresholds, respectively. Odds ratios are shown compared to clean baseline conditions. The data set contained a relatively small number of clearing swallows (*n* = 209) compared to the other two swallow types, and there were no occurrences of penetration–aspiration for clearing swallows of moderately or extremely thick liquids. Preswallow residue exceeded the 1%(C2-4)^2^ threshold for 20% of the thin and 11% of the mildly thick clearing swallows. In these cases, the odds of penetration–aspiration increased 4.60-fold for thin liquids and 2.11-fold for mildly thick liquids. Results using the > 3%(C2-4)^2^ threshold were very similar, with odds ratios of 4.20 and 2.26, respectively, compared to clean baseline swallows.

**Table 3.** Frequencies of penetration–aspiration by swallow type and consistency.

Swallow type	Consistency	PAS < 3	PAS ≥ 3	Total
Clean baseline	Thin	1219 (95%)	64 (5%)	1283
	Mildly thick	436 (95%)	23 (5%)	459
	Moderately thick	374 (99%)	4 (1%)	378
	Extremely thick	417 (99%)	4 (1%)	421
	Total	2,446 (96%)	95 (4%)	2,541
Clearing swallows	Thin	101 (82%)	22 (18%)	123
	Mildly thick	46 (90%)	5 (10%)	51
	Moderately thick	21 (100%)	0 (0%)	21
	Extremely thick	14 (100%)	0 (0%)	14
	Total	182 (87%)	27 (13%)	209
Swallows of additional material	Thin	591 (92%)	53 (8%)	644
	Mildly thick	412 (92%)	37 (8%)	449
	Moderately thick	308 (96%)	12 (4%)	320
	Extremely thick	302 (98%)	7 (2%)	309
	Total	1,622 (94%)	109 (6%)	1,722

*Note.* PAS = Penetration–Aspiration Scale (Rosenbek et al., 1996).

**Table 4.** Frequencies of penetration–aspiration and odds ratios by swallow type, residue severity threshold, and consistency.

Type of swallow	Consistency	Threshold, %(C2-4)^2^	Frequency of PAS ≥ 3, *n* (%)	*OR*	95% CI	
					Lower bound	Upper bound
Clearing	Thin	> 1.0	22 (20)	4.60	2.71	7.82
		> 3.0	15 (18)	4.20	2.28	7.76
	Mildly thick	> 1.0	5 (11)	2.11	0.69	6.42
		> 3.0	4 (10)	2.26	0.82	6.24
	Moderately thick	> 1.0	0 (0)	Insufficient data		
		> 3.0	0 (0)			
	Extremely thick	> 1.0	0 (0)	Insufficient data		
		> 3.0	0 (0)			
Additional material	Thin	> 1.0	39 (9)	1.86	1.23	2.82
		> 3.0	20 (11)	2.31	1.36	3.91
	Mildly thick	> 1.0	33 (10)	2.07	1.19	3.60
		> 3.0	20 (12)	2.53	1.35	4.73
	Moderately thick	> 1.0	9 (4)	3.72	1.13	12.23
		> 3.0	4 (3)	3.14	0.77	12.76
	Extremely thick	> 1.0	4 (2)	1.99	0.49	8.02
		> 3.0	2 (2)	2.15	0.39	11.90

*Note.* PAS = Penetration–Aspiration Scale (Rosenbek et al., 1996).

For the swallows of additional material, penetration–aspiration was seen on 9% of the thin and 10% of the mildly thick swallows with preswallow residue > 1%(C2-4)^2^, with odds ratios compared to clean baseline swallows of 1.86 and 2.07, respectively. When the > 3%(C2-4)^2^ threshold was used, the odds ratios increased to 2.31 and 2.53. With the moderately and extremely thick liquids, frequencies of unsafe swallows were 4% and 2%, respectively, when preswallow residue fell above the 1%(C2-4)^2^ threshold, and 3% and 2%, respectively, above the 3%(C2-4)^2^ threshold. Corresponding odds ratios showed a 1.99- to 3.72-fold increase in the risk of unsafe swallows, compared to clean baseline swallows.

Finally, Table 5 shows frequencies of penetration–aspiration and odds ratios (compared to clean baseline swallows) for swallows of thin liquid where preswallow residue was present but fell below 1%(C2-4)^2^. Here, there were insufficient data available to explore clearing swallows or to explore swallows of additional material with mildly, moderately, or extremely thick consistencies. On swallows of additional material with thin liquids that occurred in the context of small amounts of preswallow residue, the odds of penetration–aspiration began to exceed 1.0 at the 0.5%(C2-4)^2^ threshold.

**Table 5.** Frequencies and odds ratios for penetration–aspiration on thin liquid swallows with preswallow residue below 1%(C2–4)^2^.

Type of swallow	Threshold, %(C2-4)^2^	Frequency of PAS ≥ 3, *n* (%)	*OR*	95% CI	
				Lower bound	Upper bound
Clearing	< 1.0	0 (0)	Insufficient data		
Additional material	< 0.45	3 (5)	0.95	0.29	3.12
	< 0.5	6 (8)	1.61	0.67	3.84
	< 1.0	14 (7)	1.39	0.76	2.53

*Note.* PAS = Penetration–Aspiration Scale (Rosenbek et al., 1996).

## Discussion

Previous studies of residue-related risk of airway invasion have been limited either to clearing swallows only (Molfenter & Steele, 2013) or combined examples of clearing swallows with swallows of additional material (Namasivayam-MacDonald & Riquelme, 2019). In this study, we explored clean baseline, clearing swallows, and swallows of additional material separately. The large number of swallows in the data set that was analyzed for this article provides an opportunity to gain greater insight regarding the risk of penetration–aspiration related to residue that is already present in the pharynx at the beginning of a new swallow.

It is important to recognize that preswallow residue is only one of a number of possible mechanisms leading to airway invasion. In this data set, we observed penetration–aspiration on 5% of the thin and mildly thick liquid swallows and 1% of the moderately and extremely thick swallows with clean baseline conditions, in which preswallow residue did not play a role. In addition to pathophysiological mechanisms, such as incomplete or mistimed laryngeal vestibule closure (Curtis et al., 2020; Vose & Humbert, 2019), one known risk for penetration–aspiration, which we are unable to quantify based on videofluoroscopy, is the presence of pooled secretions in the pharynx prior to a swallow (Miles et al., 2018; Murray et al., 1996). Future studies involving simultaneous videofluoroscopy and endoscopy would be needed to elucidate this issue.

### Clearing Swallows

This data set contained 209 clearing swallows, distributed across the four consistencies studied. For clearing swallows with preswallow residue of > 1%(C2-4)^2^, the risk of penetration–aspiration compared to clean baseline swallows was 4.6-fold greater with thin liquid and 2.11-fold greater with mildly thick liquids. Thus, an important take-home message for clinicians is that risk of penetration–aspiration is present on clearing swallows of thin and mildly thick liquid.

It may initially seem counterintuitive that penetration–aspiration was more common on clearing swallows than on swallows of additional material. Here, it is important to remember that the data set contained far fewer examples of clearing swallows than swallows of additional material (*n* = 209 vs. 1,722). Furthermore, the starting conditions of clearing swallows were worse, with respect to the amount of preswallow residue present (range: 0.31–23.52%(C2-4)^2^ vs. 0.06–32.09%(C2-4)^2^), and more than 90% of the clearing swallows of thin and mildly thick liquids began with preswallow residue above the 1%(C2-4)^2^ threshold.

In this data set, we saw no penetration–aspiration on clearing swallows of moderately and extremely thick liquids. It is tempting to speculate that the thicker consistencies of these stimuli might make penetration–aspiration on clearing swallows less likely. However, it must be remembered that, in total, this data set contained only 35 examples of clearing swallows for the moderately and extremely thick consistencies, of which the strong majority (95% and 72%, respectively) had preswallow residue of > 1%(C2-4)^2^. Additionally, the small number of clearing swallows in the data set, overall, means that the study was underpowered to parse out the relative risk of penetration–aspiration on clearing swallows with residue in the valleculae versus the pyriform sinuses.

### Swallows of Additional Material

In comparison to clean baseline swallows, this study showed a 1.86- to 3.72-fold increased risk of penetration–aspiration on swallows of additional material with preswallow residue above the 1%(C2-4)^2^ threshold, depending on the consistency of the new material. With preswallow residue above the 3%(C2-4)^2^ threshold, the risk was 2.15- to 3.14-fold. Here, however, caution is warranted due to the small number of available data points above the higher threshold, which leads to less precise estimates of the odds, in the form of wider CIs (see Table 4).

With thin and mildly thick liquids, penetration–aspiration was seen on at least 9% of the swallows of additional material. Compared to thin and mildly thick liquids, the data set showed lower frequencies of penetration–aspiration on swallows of additional material with moderately and extremely thick liquids. The study did not include slightly thick liquids. Although it is tempting to speculate that the consistencies of the moderately and extremely thick boluses may have lowered the risk of airway invasion, it should be noted that smaller bolus volumes on these stimuli (which were delivered by teaspoon rather than sipped from a cup) may be a confounding factor.

The finding that penetration–aspiration was observed in 9% of the swallows of new thin liquid boluses with preswallow residue of > 1%(C2-4)^2^ is of clinical concern, because it suggests that the relatively common practice of recommending thin liquid washes to clear residue may involve risk of airway invasion. As previously explained, the consistency label for the swallows of additional material in this data set reflects the consistency of the new material that was being swallowed. Except in the case of clearing swallows (where the consistency of the preceding swallow was known), we did not attempt to identify the consistency of preswallow residue, given that it might comprise residue that had accumulated across any one or more of the preceding boluses in the study protocol. However, given the fixed order of bolus presentation in the study, the consistency of preswallow residue can be presumed to have been thin for all of the additional swallows of thin liquid. Consequently, this study does not specifically speak to the risk of penetration–aspiration when a thin liquid wash is being used to try to clear residue of thicker consistencies.

### Thresholds

The preswallow residue thresholds selected for examination in this analysis were guided by information regarding the distribution of postswallow pharyngeal residue in healthy adults (Steele, Peladeau-Pigeon, et al., 2019). Although the data point to preswallow residue above 1%(C2-4)^2^ carrying risk of penetration–aspiration, we cannot claim to have found the true lower boundary at which this risk emerges. As shown in Table 5, the data set contained 14 swallows of additional thin liquid material for which preswallow residue was judged to be present but fell below the 1%(C2-4)^2^ threshold. Based on exploration of this very small sample, increased risk of penetration–aspiration appeared to emerge above a value of 0.5%(C2-4)^2^. There was insufficient data available to explore this question with the thicker consistencies. Further study with larger data sets is required to confirm where the boundary between “safe” versus risky residue lies.

### Issues Regarding Definitions

Like the original study from which the data were taken (Steele, Mukherjee, et al., 2019) and like previous studies of residue-related risk of airway invasion (Molfenter & Steele, 2013; Namasivayam-MacDonald & Riquelme, 2019), this analysis used a binary reduction of the PAS (< vs. ≥ 3) to denote “safe” versus “unsafe” swallows. It should be noted that 515 of the swallows in the data set had PAS scores of 2; although these swallows were included in the “safe” class in this analysis, they do involve transient penetration of material into the upper laryngeal vestibule. Conversely, 14 swallows included in the “unsafe” class had PAS scores of 4, which represents deeper penetration, but an ultimate outcome with no material remaining in the airway. Future studies might consider alternative approaches to classifying airway invasion, such as the categorical approach proposed by Steele and Grace-Martin (2017). Notably, the data set for this study contained no examples of PAS scores of 6, representing entry of material below the true vocal folds with subsequent ejection.

With respect to measuring residue, this study adopted a relatively new metric, in which residue area is expressed as a percentage of the (C2-4)^2^ anatomical reference area. This choice has the disadvantage of making it impossible to compare the results directly to previous studies using the NRRS. However, an advantage is the opportunity to add residue measures from the valleculae and pyriform sinuses together to yield a composite score (Steele et al., 2020). It is interesting to note that the baseline residue seen in this data set was rarely isolated to the pyriform sinuses, such that estimates of risk related to residue in the pyriform sinuses could not be extrapolated separately from the risks associated with residue in other locations.

## Conclusions

The challenge of establishing residue thresholds that predict safe versus unsafe subsequent swallows is not trivial. In this study, we adopted thresholds of 1% and 3%(C2-4)^2^ based on reference data from a recent study of swallowing in healthy adults (Steele, Peladeau-Pigeon, et al., 2019). These thresholds represent the 75th and 95th percentiles of residue distribution on thin liquids in that study and, as such, are analogous to the cut-points that are conventionally used in medicine to identify situations approaching or falling outside the limits of the “normal” range in blood test lab values (Ceriotti et al., 2009; Ozarda, 2016; Peck & Olsen, 2014, Chapter 3, p. 156). Our analysis suggests that this approach is effective for delineating situations where preswallow pharyngeal residue introduces added risk for airway invasion compared to clean baseline swallow conditions. Clinicians should be aware that any swallow that is performed in the context of preswallow residue of > 1%(C2-4)^2^ involves a heightened risk of penetration–aspiration. This result establishes a compelling need to develop and investigate the impact of interventions targeting residue prevention or reduction, and routine measurement of residue, in addition to penetration–aspiration as an outcome of dysphagia treatment, is strongly recommended.

## Author Contributions

**Catriona M. Steele:** Conceptualization (Lead), Data curation (Supporting), Formal analysis (Lead), Funding acquisition (Lead), Investigation (Lead), Methodology (Equal), Supervision (Lead), Writing - Original Draft (Lead), Writing - Review & Editing (Lead); principal investigator for the project and was responsible for project design, statistical analysis, and article writing. **Melanie Peladeau-Pigeon:** Data curation (Lead), Methodology (Supporting), Project administration (Lead), Supervision (Supporting), Writing - Review & Editing (Supporting); managed data processing and compiled all the videofluoroscopy ratings for this project, as well as contributing to article editing. **Emily Barrett:** Data curation (Equal), Writing - Review & Editing (Equal); completed the majority of the videofluoroscopy rating for this article and contributed to article editing. **Talia S. Wolkin:** Data curation (Equal), Writing - Review & Editing (Equal); completed the majority of the videofluoroscopy rating for this article and contributed to article editing.
